# SPA-QNAS: Improving Search Efficiency and Stability in Evolutionary Quantum Neural Architecture Search

**DOI:** 10.3390/e28070829

**Published:** 2026-07-22

**Authors:** Linwei Shang, Hao Cao, Yang Wu, Xufeng Niu, Junjie Chen

**Affiliations:** 1 College of Information and Network Engineering, Anhui Science and Technology University, Bengbu 233000, China; lwhyx_1@163.com (L.S.); wuyang20030806@163.com (Y.W.); yjs2024124@ahstu.edu.cn (J.C.); 2 School of Computer Science and Engineering, Shaanxi Key Laboratory for Network Computing and Security Technology, Xi’an University of Technology, Xi’an 710048, China; xufeng_niu@xaut.edu.cn

**Keywords:** quantum neural architecture search, quantum evolutionary algorithm, structural probability enhancement, adaptive evolutionary control

## Abstract

Quantum neural architecture search (QNAS) has emerged as a promising approach for automatically designing parameterized quantum circuits (PQCs) for near-term quantum machine learning tasks. However, quantum evolutionary algorithm (QEA)-based QNAS methods often suffer from slow distribution concentration and insufficient update adaptivity in high-dimensional discrete search spaces, which limits both search efficiency and final model performance. To address these issues, this paper proposes Structural Probability Adaptive Quantum Neural Architecture Search (SPA-QNAS), a search-dynamics-enhanced QNAS method built upon the EQNAS benchmark framework. SPA-QNAS introduces two complementary mechanisms into the QPV-driven evolutionary search loop: Structural Probability Enhancement (SPE) and Adaptive Evolutionary Control (AEC). SPE reinforces elite structural decisions to accelerate the concentration of the structural sampling distribution toward high-fitness regions, while AEC adaptively regulates the rotation updates of non-elite individuals according to fitness feedback, thereby improving update stability and suppressing ineffective disturbances. Under the same search space, circuit template, and quantum resource budget as EQNAS, SPA-QNAS is evaluated on the MNIST and Warship benchmark datasets. Experimental results across multiple independent runs demonstrate that SPA-QNAS achieves higher classification accuracy and more stable performance compared with EQNAS. In representative experiments, SPA-QNAS achieves classification accuracies of 99.42% on MNIST and 85.33% on Warship under the same search space and quantum resource budget as EQNAS. These results indicate that improving QPV-based evolutionary update dynamics is an effective way to enhance the stability and robustness of QNAS under fixed quantum resource constraints.

## 1. Introduction

Quantum neural networks (QNNs) commonly employ parameterized quantum circuits (PQCs) as an important modeling paradigm [1,2], where trainable quantum gates are used for feature representation and discriminative modeling [3,4,5,6]. In recent years, PQC-based QNNs have been increasingly applied to image classification and related tasks, showing potential application value [7,8]. However, under noisy intermediate-scale quantum (NISQ) conditions, the number of quantum gates, circuit depth, and the scale of trainable parameters can affect model trainability, robustness, and potential deployability [5,9]. In particular, overly deep or improperly parameterized PQCs may suffer from vanishing gradients or the barren plateau phenomenon, thereby increasing the difficulty of model optimization [10,11]. Since the gate types, connection patterns, and parameter scale of a PQC directly affect model expressiveness and resource consumption [12], relying solely on manual experience to design quantum circuits often makes it difficult to achieve a stable balance between performance and complexity. Therefore, automatically designing high-performance and compact quantum circuits under limited quantum resource constraints has become an important problem in quantum machine learning.

To reduce the dependence of quantum circuit design on manual expertise, quantum neural architecture search (QNAS) introduces automated search strategies from classical neural architecture search (CNAS), such as reinforcement-learning-based search [13], evolutionary search [14], and differentiable search [15], into quantum circuit optimization; these strategies have been widely studied in the NAS literature [16]. In this context, QNAS generates candidate quantum circuit structures within a predefined search space and selects better-performing architectures according to evaluation metrics [17,18]. In recent years, QNAS has developed along multiple technical directions, including noise-aware supernet search, differentiable search, staged tree search, representation learning-assisted search, and evolutionary search [6,17,18,19,20,21,22,23,24]. Among these directions, evolutionary QNAS searches discrete quantum circuit structures through probabilistic encoding, candidate-structure sampling, fitness evaluation, and iterative updates, making it suitable for handling high-dimensional discrete search spaces formed by combinations of candidate gates [19,25,26].

The performance of evolutionary QNAS is highly dependent on its search dynamics. When candidate structures repeatedly undergo sampling, training, evaluation, and updating, search efficiency depends on whether the sampling distribution can rapidly concentrate toward high-fitness regions, while search stability depends on whether the update rule can maintain a proper balance between exploration and exploitation. As a representative evolutionary QNAS method, EQNAS adopts a quantum evolutionary algorithm (QEA)-based search strategy and uses quantum-inspired probability-amplitude vectors (QPVs) to probabilistically encode candidate quantum circuit structures [25,26]. Under the EQNAS benchmark setting, the candidate gate set {XX,YY,ZZ,I} forms a high-dimensional discrete architecture search space, and architecture search is performed through observation-based sampling, training-based evaluation, and rotation updates [25]. Existing experiments using EQNAS have verified the feasibility of evolutionary QNAS for image classification tasks; however, from the perspective of search dynamics, its QPV update mechanism still leaves room for further improvement.

Specifically, this room for improvement is mainly reflected in two aspects. First, elite structural information is mainly propagated to the sampling distribution gradually through iterative rotation updates, without an explicit mechanism for enhancing the sampling probabilities of high-fitness regions. As a result, in high-dimensional discrete search spaces, the sampling distribution may concentrate toward high-fitness regions relatively slowly [25,26]. Second, the fixed-step rotation update rule does not explicitly distinguish the effects of non-elite individuals with different fitness levels on the evolution of the probability distribution. Therefore, it may lack sufficient adaptivity across different evolutionary stages and may introduce ineffective disturbances, thereby affecting search stability and final performance [25,26].

Based on the above analysis, this paper proposes Structural Probability Adaptive Quantum Neural Architecture Search (SPA-QNAS), an enhanced evolutionary quantum neural architecture search method built upon the EQNAS benchmark framework. SPA-QNAS introduces two complementary mechanisms into the QPV-driven evolutionary search loop, namely Structural Probability Enhancement (SPE) and Adaptive Evolutionary Control (AEC). SPE applies bounded probability reinforcement to the key decision positions of the contemporary elite structure, thereby accelerating the concentration of the sampling distribution toward high-fitness regions. AEC adaptively adjusts the rotation update strength of non-elite individuals according to fitness feedback, suppressing ineffective disturbances from low-quality individuals and improving update stability.

Unlike approaches that obtain performance improvements by modifying the circuit template, enlarging the search space, or increasing quantum resources, SPA-QNAS focuses on enhancing the search dynamics of evolutionary QNAS under the same benchmark setting as EQNAS. Specifically, the candidate gate set, circuit template, training configuration, and resource budget are kept unchanged, while the proposed SPE and AEC mechanisms are introduced to improve elite-guided structural probability reinforcement and adaptive non-elite QPV alignment. The main contributions of this paper can be summarized as follows:

1. An enhanced evolutionary quantum neural architecture search method, SPA-QNAS, is proposed based on the EQNAS benchmark framework. The proposed method focuses on enhancing the search dynamics of the QPV-driven search process, improving search efficiency and update stability without changing the original search space or resource budget.
2. An SPE mechanism is proposed. This mechanism applies bounded probability reinforcement to the key decision positions of the contemporary elite structure, explicitly increasing the sampling probabilities of high-fitness regions. In this way, it accelerates the concentration of the structural sampling distribution toward high-fitness regions while preserving necessary search diversity through probability bounds.
3. An AEC mechanism is proposed. This mechanism adaptively adjusts the QPV rotation update strength according to the fitness gap between each non-elite individual and the contemporary elite, thereby alleviating ineffective disturbances caused by fixed-step updates and improving the stability of the evolutionary search process.
4. Experiments are conducted under the same search space, training configuration, and fixed quantum resource budget as the EQNAS benchmark [25]. The results show that SPA-QNAS can simultaneously improve search efficiency and final classification performance without increasing the number of qubits, quantum gates, or trainable parameters.

For clarity and readability, the main abbreviations and acronyms used throughout this paper are summarized in Table 1.

The remainder of this paper is organized as follows. Section 2 introduces the theoretical background and the EQNAS benchmark framework. Section 3 presents the proposed SPA-QNAS methodology, including the SPE and AEC mechanisms. Section 4 describes the experimental settings and presents the comparative evaluation of SPA-QNAS. Section 5 concludes the paper and discusses future research directions.

## 2. Preliminaries

This section introduces the preliminary concepts directly related to the proposed method. We first briefly describe the PQC modeling setting adopted in this work, and then present the EQNAS benchmark framework, which provides the necessary background for the design and experimental analysis of SPA-QNAS.

### 2.1. Parameterized Quantum Circuit Modeling Background

The quantum neural architecture studied in this paper is built upon the standard PQC paradigm. In general, a PQC-based quantum neural network consists of three main stages: data encoding, trainable parameterized quantum circuit transformation, and measurement readout [27]. First, a classical input sample is mapped to a quantum state through a fixed encoding module. Then, the quantum circuit transforms the encoded quantum state through trainable parameterized quantum gates and candidate quantum operations to perform feature representation and discriminative modeling. Finally, the classification output is obtained by measuring the readout qubit [25].

A single-qubit state can be represented as |ψ〉=α|0〉+β|1〉, where α and β satisfy the normalization constraint ${\left|\alpha \right|}^{2}+{\left|\beta \right|}^{2}=1$.

In quantum neural networks, a multi-qubit system is constructed from single qubits through tensor products, and quantum superposition and entanglement provide the basis for parallel state representation. For a parameterized quantum circuit, the overall circuit transformation can be abstractly expressed as:

(1) $$U\left(\theta \right)=U_{L}\left(\theta_{L}\right)U_{L-1}\left(\theta_{L-1}\right)\cdots U_{1}\left(\theta_{1}\right)$$

where U(θ) denotes the circuit transformation composed of multiple layers of parameterized quantum gates, and θ denotes the set of continuous trainable parameters. Given an input quantum state |z,1〉, the output state after passing through the parameterized quantum circuit can be written as:

(2) $$|\Psi^{\prime }\rangle =U\left(\theta \right)\;|z,1\rangle$$

Here, |z〉 denotes the encoded input data quantum state, and the additional readout qubit is used for subsequent classification measurement.

In this paper, architecture search is performed on the discrete structural topology of the PQC, while the continuous gate parameters are optimized after a candidate structure has been sampled and determined. Specifically, the parameters θ are initialized and iteratively updated through a classical optimization procedure. At each optimization step, the parameterized circuit is executed to obtain the measurement output. The corresponding loss is then calculated on the training data, and the parameters are updated according to the optimization rule. In the experiments of this work, Adam is adopted as the classical optimizer for continuous parameter training.

Therefore, the PQC provides the fixed quantum modeling basis for evaluating candidate architectures. Under this fixed setting, this work focuses on improving the search dynamics of evolutionary quantum neural architecture search.

### 2.2. Benchmark Framework: Evolutionary Quantum Neural Architecture Search (EQNAS)

This paper adopts EQNAS as the benchmark framework. EQNAS is an evolutionary quantum neural architecture search method for image classification tasks. It adopts the quantum-inspired evolutionary algorithm (QEA) as the search strategy and uses probability-encoded quantum chromosomes, namely, to represent the structural sampling distribution of candidate architectures [25,26].

**Figure 1:**
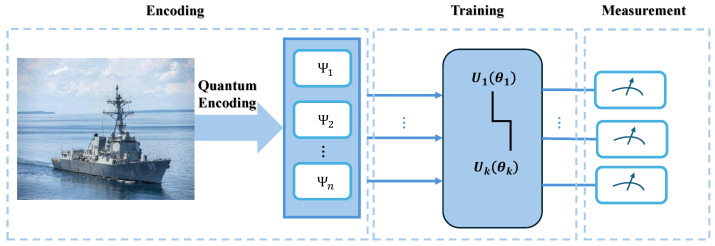
Schematic illustration of the basic QNN architecture adopted in the EQNAS benchmark.

In EQNAS, the quantum neural network consists of three stages: encoding, parameterized circuit transformation, and measurement. First, the input image is mapped to a quantum state through qubit-lattice encoding [28]. Then, in the parameterized circuit transformation stage, the circuit performs feature extraction and discriminative modeling through multiple layers of trainable single-qubit rotation gates and two-qubit entangling gates [25]. Finally, the readout qubit is measured to obtain the classification result [25]. Following the original benchmark setting, EQNAS uses 16 data qubits and one readout qubit, resulting in a 17-qubit quantum neural network [25]. The basic QNN architecture of the EQNAS benchmark is shown in Figure 1.

In terms of architecture representation, EQNAS adopts the quantum chromosome representation from the quantum-inspired evolutionary algorithm (QEA) framework [26] to encode candidate quantum circuit architectures. Each candidate position is assigned one operation from the candidate gate set {XX,YY,ZZ,I}, where XX, YY, and ZZ denote parameterized two-qubit entangling operations, and *I* denotes an identity operation corresponding to no active gate operation at that position.

**Figure 2:**
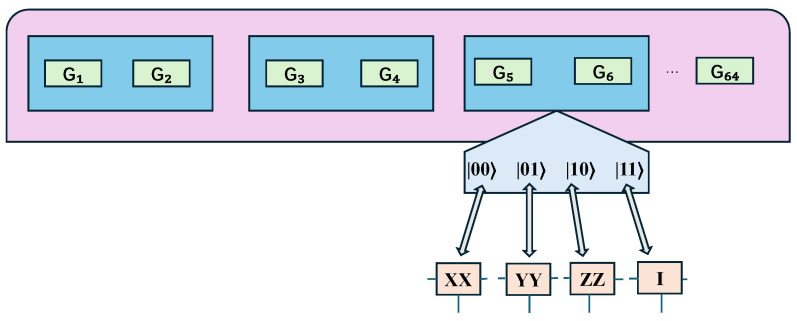
Quantum chromosome encoding in EQNAS.

According to the EQNAS encoding scheme, each candidate gate is represented by two binary genes, and the four binary patterns correspond to the four candidate operations, as shown in Figure 2. The entire search space contains 32 candidate positions, and each position has four possible gate choices. Therefore, the corresponding discrete architecture search space has a size of $4^{32}$, which is one of the benchmark settings adopted in this paper.

Finally, the quantum-inspired probability-amplitude vectors (QPVs) are iteratively updated through rotation-gate update rules, interference crossover operators, and elite preservation, thereby shaping the structural sampling distribution toward promising regions. The encoded quantum chromosomes are then decoded through observation to generate deterministic candidate architectures. For each sampled architecture, the corresponding parameterized quantum circuit is constructed and trained on a quantum simulator, and its classification performance is used as the fitness value for subsequent evolutionary updates.

At the implementation level, the original EQNAS paper provides two software stacks: one based on Cirq + TensorFlow Quantum (TFQ), and the other based on MindQuantum. The original experimental results show that EQNAS can improve classification accuracy over manually designed QNNs on the MNIST and Warship datasets, and can reduce the number of qubits, quantum gates, or trainable parameters in some experimental settings. These results demonstrate its representativeness as an evolutionary quantum neural architecture optimization benchmark.

This paper selects EQNAS as the benchmark framework for three main reasons. First, EQNAS is a representative discrete evolutionary QNAS method that adopts QEA as the search strategy and QPVs as the probabilistic encoding representation. Second, its search space, candidate gate set, circuit template, and resource budget are clearly defined, which facilitates strictly consistent comparative experiments. Third, the modifications introduced in this work are limited to the search dynamics themselves, namely the enhanced update of the structural sampling distribution and the adaptive control of non-elite QPV rotation updates, without changing the basic task setting or overall resource constraints of EQNAS.

Therefore, in SPA-QNAS, the search space, candidate gate set, circuit template, and overall quantum resource budget are kept consistent with EQNAS. The main objective of this paper is to specifically enhance the search efficiency and update stability of QPV-driven evolutionary QNAS under the same EQNAS benchmark setting.

## 3. Method

Building upon the EQNAS benchmark framework, this paper proposes SPA-QNAS, an enhanced evolutionary quantum neural architecture search method designed to improve search efficiency and update stability in large discrete search spaces. SPA-QNAS introduces two complementary mechanisms into the QEA-based search loop: Structural Probability Enhancement (SPE) and Adaptive Evolutionary Control (AEC).

In the present implementation, the structural sampling distribution is implicitly represented by the collection of quantum-inspired probability-amplitude vectors (QPVs) across the population, rather than by a single global probabilistic model. Each individual maintains its own QPV, and the population as a whole induces a stochastic sampling distribution over candidate circuit structures. Under this formulation, updating individual QPVs corresponds to shaping the overall sampling distribution at the population level.

**Figure 3:**
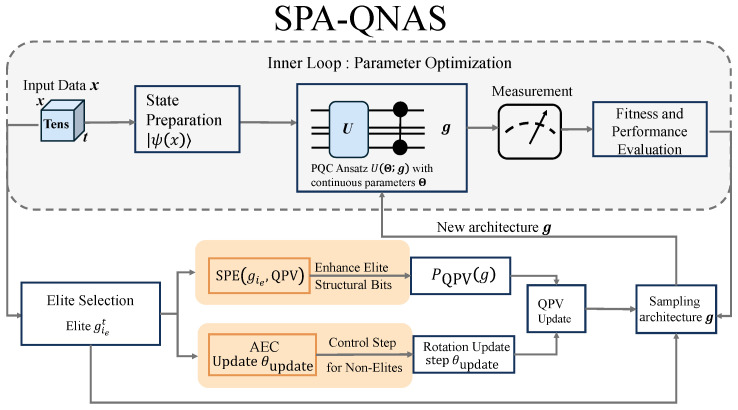
Schematic diagram of the evolutionary search workflow of SPA-QNAS. The inner loop performs parameter optimization and fitness evaluation of sampled candidate circuits, while the outer loop updates the QPV-based structural sampling process through SPE-based elite probability enhancement and AEC-based adaptive rotation control.

Within this framework, SPE explicitly reinforces the probability amplitudes of the contemporary elite individual, thereby strengthening high-fitness regions in the population. AEC, in contrast, adaptively adjusts the rotation-update strength of non-elite individuals based on their fitness gap with respect to the elite, progressively aligning the population toward high-fitness regions. Together, SPE biases the sampling distribution toward promising regions, while AEC regulates the convergence dynamics to ensure stable and gradual propagation of elite structural information across the population. Figure 3 illustrates the overall workflow of SPA-QNAS. The inner loop performs candidate-circuit training and evaluation, while the outer loop updates the population-level QPV-based sampling distribution via SPE and AEC.

To provide a unified description of structure search and parameter training, the overall process is formulated as a two-stage optimization problem. Let G denote the discrete search space of candidate circuit topologies, and let g∈G denote a sampled candidate structure. For any sampled structure *g*, its continuous gate parameters are obtained by minimizing the training loss on the training set:

(3) $$\theta^{*}\left(g\right)=\arg\min_{\theta }L\left(g,\theta ;D_{\mathrm{train}}\right)$$

Its validation performance is then used as the fitness:

(4) $$F\left(g\right)=\mathrm{Evaluate}\left(g,\theta^{*}\left(g\right);D_{\mathrm{val}}\right),\;g^{\mathrm{best}}=\arg\max_{g\in G}F\left(g\right)$$

where θ denotes the continuous gate parameters trained by a classical optimizer after the structure is determined, and $D_{\mathrm{train}}$ and $D_{\mathrm{val}}$ denote the training and validation datasets, respectively.

### 3.1. Structural Probability Enhancement for the Structural Sampling Distribution

In QPV-driven evolutionary QNAS, the structural sampling distribution is progressively shaped through the evolution of population QPVs. However, when the search space is large and discrete, relying only on gradual population updates may cause the distribution to concentrate toward high-fitness regions relatively slowly. To address this issue, SPA-QNAS introduces the SPE strategy, which explicitly reinforces the structural decisions of the contemporary elite.

In the quantum-inspired representation adopted in this work, the quantities $\alpha_{i,j}$ and $\beta_{i,j}$ are real-valued parameters representing probability amplitudes for evolutionary encoding rather than general complex-valued quantum wavefunction coefficients. For an individual *i* at binary gene position *j*, let the probability amplitudes be denoted by $\left(\alpha_{i,j},\beta_{i,j}\right)$, with the corresponding sampling probabilities:

(5) $$P_{i,j}\left(0\right)=\alpha_{i,j}^{2},\;P_{i,j}\left(1\right)=\beta_{i,j}^{2}$$

subject to the normalization constraint:

(6) $$\alpha_{i,j}^{2}+\beta_{i,j}^{2}=1$$

At initialization, all genes are set to the standard uniform probability-amplitude state:

(7) $$\alpha_{i,j}=\beta_{i,j}=\frac{1}{\sqrt{2}}$$

so that $P_{i,j}\left(0\right)=P_{i,j}\left(1\right)=0.5$. This provides a neutral starting point for structure search.

After fitness evaluation in generation *t*, the current elite individual $i_{e}$ is identified. For each binary gene position *j*, let the observed elite bit be $x={\left(g_{i_{e}}\right)}_{j}\in \left\{0,1\right\}$. SPE explicitly reinforces the probability of the elite bit as:

(8) $$P_{i_{e},j}\left(x\right)\leftarrow \mathrm{Clip}\left(P_{i_{e},j}\left(x\right)+\delta ,\;\left[p_{\min},p_{\max}\right]\right)$$

where δ is the enhancement step size, $\left[p_{\min},p_{\max}\right]$ is the clipping interval, and Clip(·) prevents the distribution from degenerating into a deterministic state. The complementary probability is updated by:

(9) $$P_{i_{e},j}\left(1-x\right)=1-P_{i_{e},j}\left(x\right)$$

where $P_{i_{e},j}\left(1-x\right)$ denotes the complementary probability associated with the elite bit *x*. Since the two probabilities form a normalized binary distribution, the complementary probability is determined through binary probability normalization rather than any physical quantum operation. After reinforcement, the elite probability amplitudes are reconstructed as:

(10) $$\alpha_{i_{e},j}=\sqrt{P_{i_{e},j}\left(0\right)},\;\beta_{i_{e},j}=\sqrt{P_{i_{e},j}\left(1\right)}$$

Therefore, SPE acts as an explicit *elite-only probability shaping* step. It strengthens the probability representation of elite structural decisions in the QPV-based sampling distribution while preserving search diversity through bounded probability clipping. The updated probability amplitudes define the structural sampling distribution for the next generation, from which new candidate architectures are stochastically generated. A more general analysis of how SPE affects probability concentration is provided in the following mechanism-level discussion.

### 3.2. Adaptive Evolutionary Control for Non-Elite Quantum-Inspired Probability-Amplitude Vector Rotation Updates

While SPE biases the structural sampling distribution toward promising regions, the population still requires a stable and adaptive alignment mechanism to propagate elite structural information to non-elite individuals. To this end, SPA-QNAS introduces an AEC mechanism at the QPV rotation-update layer.

In the baseline QEA-based update rule, non-elite individuals are updated using a fixed rotation step size. Such a fixed update rule may be inadequate across different evolutionary stages, since the required adjustment strength varies with the fitness gap between the current individual and the elite. In the present implementation, AEC replaces the fixed-step update with a fitness-gap-based adaptive rotation rule.

In this work, search stability is defined from two complementary aspects. First, intra-run stability refers to the ability of the evolutionary update process to avoid abrupt and ineffective perturbations during a single search run, so that the QPV-based structural sampling distribution evolves smoothly rather than being dominated by unstable updates from low-quality individuals. Second, inter-run stability refers to the robustness of the search outcome across independent runs with different random seeds, which is reflected by the standard deviation of the final classification accuracy.

For a non-elite individual *i*, let $F_{i_{e}}$ denote the fitness of the contemporary elite individual and $F_{i}$ denote the fitness of individual *i*. The normalized fitness-gap ratio is defined as:

(11) $$r_{i}=\mathrm{Clip}\;\left(\frac{F_{i_{e}}-F_{i}}{\left|F_{i_{e}}\right|+\epsilon },\;0,1\right)$$

where ϵ is a small positive constant introduced for numerical stability. Based on this ratio, the adaptive rotation magnitude is computed as:

(12) $$\Delta \theta_{i}=\theta_{\min}+\left(\theta_{\max}-\theta_{\min}\right)\;r_{i}$$

where $\theta_{\min}$ and $\theta_{\max}$ denote the minimum and maximum rotation angles, respectively.

For binary gene position *j*, if the current bit already matches the elite bit, no update is performed. Otherwise, the update direction is determined by the elite bit:

(13) $$\theta_{i,j}=\left\{\begin{array}{cc} 0, & {\left(g_{i}\right)}_{j}={\left(g_{i_{e}}\right)}_{j},\\ -\Delta \theta_{i}, & {\left(g_{i}\right)}_{j}=1,\;{\left(g_{i_{e}}\right)}_{j}=0,\\ +\Delta \theta_{i}, & {\left(g_{i}\right)}_{j}=0,\;{\left(g_{i_{e}}\right)}_{j}=1. \end{array}\right.$$

For clarity, the corresponding direction rules are summarized in Table 2.

Then, the probability amplitudes of the non-elite individual at this position are updated using a rotation matrix:

(14) $$\left[\begin{array}{c} \alpha_{i,j}^{t+1}\\ \beta_{i,j}^{t+1} \end{array}\right]=\left[\begin{array}{cc} \cos\theta_{i,j} & -\sin\theta_{i,j}\\ \sin\theta_{i,j} & \cos\theta_{i,j} \end{array}\right]\left[\begin{array}{c} \alpha_{i,j}^{t}\\ \beta_{i,j}^{t} \end{array}\right]$$

In this quantum-inspired QPV representation, the rotation matrix acts as a norm-preserving transformation that modifies the probability amplitudes rather than the observed binary genes. By geometrically adjusting the probability-amplitude vector toward the elite structural pattern, the sampling probabilities are gradually modified while preserving the normalization constraint. Consequently, non-elite individuals are probabilistically aligned with promising structural patterns without eliminating the stochastic exploration capability of the population. After rotation, the updated sampling probabilities for the next generation are obtained as:

(15) $$P_{i,j}\left(0\right)={\left(\alpha_{i,j}^{t+1}\right)}^{2},\;P_{i,j}\left(1\right)={\left(\beta_{i,j}^{t+1}\right)}^{2}$$

AEC therefore provides a stable population-level alignment mechanism. Individuals with larger fitness gaps receive larger rotation updates, resulting in stronger alignment toward the elite structural pattern, while individuals already close to the elite are updated more conservatively. Under the above definition, AEC mainly improves intra-run stability by suppressing ineffective perturbations from low-quality structures and promoting smoother evolutionary dynamics. The resulting updated QPVs are then used for sampling candidate structures in the subsequent generation, completing the feedback loop between fitness evaluation and structural evolution.

### 3.3. Mechanism-Level Analysis of Search Dynamics

To further clarify how SPE and AEC affect the search behavior of the baseline QEA-based QNAS framework, this subsection provides a mechanism-level analysis of the proposed update rules. The purpose of this analysis is not to claim a strict global convergence guarantee, but to explain how the two mechanisms affect probability concentration, update stability, and computational complexity under the same evolutionary search framework.

First, SPE can be interpreted as a bounded probability-reinforcement mechanism for elite structural decisions. Consider a binary gene position *j* in the QPV-based structural encoding, and let x∈{0,1} denote the bit value selected by the contemporary elite individual at this position. According to the SPE update rule, the sampling probability of this elite bit is increased by an enhancement step size δ and then clipped into a predefined probability interval. This process can be written as

(16) $$P_{t+1,j}\left(x\right)=\mathrm{Clip}\left(P_{t,j}\left(x\right)+\delta ,\;\left[p_{\min},p_{\max}\right]\right),$$

where $P_{t,j}\left(x\right)$ denotes the sampling probability of bit value *x* at position *j* in generation *t*, and $p_{\min}$ and $p_{\max}$ denote the lower and upper probability bounds, respectively. If the same elite structural decision is repeatedly reinforced over *k* successive generations and no opposite reinforcement is applied to this position during this interval, the sampling probability follows

(17) $$P_{t+k,j}\left(x\right)=\min\left(P_{t,j}\left(x\right)+k\delta ,\;p_{\max}\right).$$

This relation shows that structural decisions repeatedly associated with high-fitness elite individuals receive progressively larger sampling probabilities until the upper probability bound is reached. Therefore, SPE provides a direct probability-shaping mechanism that promotes the concentration of the sampling distribution toward promising structural regions. Meanwhile, the clipping interval $\left[p_{\min},p_{\max}\right]$ prevents the sampling probability from collapsing to 0 or 1, so the evolutionary process still preserves residual exploration capability.

Second, AEC improves the stability of non-elite QPV updates through bounded adaptive rotation. In the proposed AEC mechanism, the rotation magnitude of a non-elite individual is determined by its normalized fitness-gap ratio. Since the normalized ratio satisfies $r_{i}\in \left[0,1\right]$, the adaptive rotation magnitude is bounded by

(18) $$\Delta \theta_{i}=\theta_{\min}+\left(\theta_{\max}-\theta_{\min}\right)r_{i},\;\Delta \theta_{i}\in \left[\theta_{\min},\theta_{\max}\right].$$

This bounded update rule prevents excessively large changes in the probability amplitudes. Individuals with larger fitness gaps from the elite receive stronger alignment updates, whereas individuals that are already closer to the elite are updated more conservatively. Thus, AEC acts as a fitness-dependent smoothing mechanism for non-elite QPV evolution, reducing abrupt or ineffective perturbations while maintaining the stochastic sampling property of the evolutionary process.

Third, the additional computational cost introduced by SPE and AEC is lightweight. Let *N* denote the population size and *L* denote the binary genome length of an individual. SPE updates only the elite QPV over the genome length, resulting in an O(L) operation per generation. AEC updates the non-elite QPVs over the population, resulting in an O(NL) operation per generation. These operations are performed at the QPV-update level and do not change the number of qubits, the candidate gate set, the PQC depth, the population size, or the number of evolutionary generations. Compared with the cost of training and evaluating sampled PQC architectures, the additional overhead introduced by SPE and AEC is negligible.

Overall, SPE and AEC do not alter the quantum circuit resource budget or the basic QEA-based search framework. Instead, they improve the structural sampling dynamics by reinforcing high-fitness structural decisions, constraining non-elite update magnitudes, and preserving bounded stochastic exploration. This mechanism-level analysis provides additional support for interpreting the empirical improvements in search efficiency and stability observed in the experiments, while a avoiding any claim of strict global convergence guarantee.

### 3.4. Quantum–Classical Optimization Workflow

**Figure 4:**
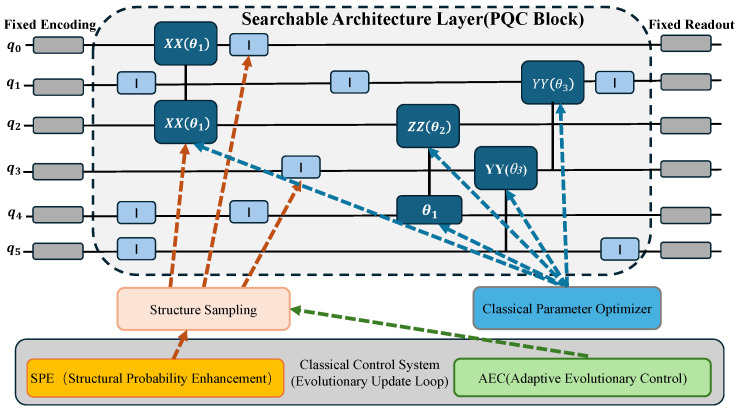
Quantum–classical optimization workflow of SPA-QNAS. The fixed encoding and readout modules remain unchanged, while the searchable PQC block is optimized through candidate-structure sampling, parameter training, and fitness-driven QPV updates with SPE and AEC. Orange and blue elements indicate structure sampling and classical parameter optimization, respectively, whereas yellow and green elements indicate SPE and AEC.

To clarify how the proposed search-dynamics enhancement interacts with PQC training and fitness evaluation, this section summarizes the quantum–classical optimization workflow used in SPA-QNAS, as shown in Figure 4.

(1) Fixed Encoding Stage

Given an input sample *x*, the fixed encoding module first maps it to a quantum-state representation. Taking the initial state ${|0\rangle }^{⊗n}$ as an example, after applying the encoding operator $U_{\mathrm{encode}}\left(x\right)$, the resulting encoded state is:

(19) $$|\psi (x)\rangle =U_{\mathrm{encode}}\left(x\right){|0\rangle }^{⊗n}.$$

This stage loads classical features into the quantum system. Its circuit structure remains fixed during architecture search and does not participate in topological evolution.

(2) Searchable Architecture Layer (Parameterized Quantum Circuit Block)

The dashed box in Figure 4 denotes the searchable parameterized quantum circuit (PQC) block. This layer contains two types of variables: a discrete structural topology *g*, composed of gates selected from the candidate set {XX,YY,ZZ,I}, and continuous trainable gate parameters Θ, which are optimized after the structure is determined.

Given a fixed topology *g*, the continuous parameters Θ inside the PQC are optimized on the training set by minimizing the loss function, yielding the structure-dependent optimal parameters $\Theta^{*}\left(g\right)$. These parameters are then used to evaluate the fitness of the current candidate structure.

For the QPVs used to control structure sampling, the classical control layer executes sequential updates after each generation’s evaluation. First, the elite individual of the current generation is identified. SPE then explicitly reinforces its binary structural decisions at the probability-amplitude level, forming a stronger elite signal in the structural sampling distribution. Next, AEC applies adaptive rotation-based updates to the probability amplitudes of non-elite individuals according to their fitness gaps with respect to the elite, guiding the population distribution toward high-fitness regions.

After circuit transformation, the readout qubit is measured in the fixed readout module, and classical post-processing is performed to obtain validation performance metrics. These metrics are fed back to the classical control system as fitness signals. Based on this feedback, the search loop performs current-generation elite selection, SPE-based elite reinforcement, AEC-based adaptive rotation updates for non-elite individuals, and mutation-based exploration. In this way, SPA-QNAS forms a closed-loop optimization process among structural sampling, parameter training, and fitness-driven QPV updates.

### 3.5. Algorithm Workflow

By integrating SPE and AEC into the EQNAS benchmark framework, the overall workflow of SPA-QNAS is summarized in Algorithm 1.
**Algorithm 1 **SPA-QNAS Evolutionary Search.**Require:** $D_{\mathrm{train}}$, $D_{\mathrm{val}}$, *N*, *L*, $G_{\max}$, *E*, $\theta_{\min}$, $\theta_{\max}$, δ, $\left[p_{\min},p_{\max}\right]$, ϵ, mutation rate $p_{m}$.**Ensure:** Global best architecture $g^{\mathrm{best}}$, optimal parameters $\Theta^{\mathrm{best}}$.  1:Initialize probability amplitudes $\left(\alpha_{i,j},\beta_{i,j}\right)$ and QPV probabilities $P_{i,j}\left(0/1\right)$; set $F^{\mathrm{best}}\leftarrow -\infty$.  2:**for **t=1 to $G_{\max}$ **do**  3:     Sample chromosomes ${\left\{g_{i}\right\}}_{i=1}^{N}$ from QPVs through stochastic observation.  4:     For each $g_{i}$: train $\Theta_{i}$ on $D_{\mathrm{train}}$ for *E* epochs; evaluate fitness $F_{i}$ on $D_{\mathrm{val}}$.  5:     Select the elite individual $i_{e}=\arg\max_{i}F_{i}$; set $g_{i_{e}}$ as the contemporary elite. If $F_{i_{e}}>F^{\mathrm{best}}$, update $\left(F^{\mathrm{best}},g^{\mathrm{best}},\Theta^{\mathrm{best}}\right)$.  6:     SPE (elite only): for each binary gene position *j*, let $x={\left(g_{i_{e}}\right)}_{j}$; update$$P_{i_{e},j}\left(x\right)\leftarrow \mathrm{Clip}\left(P_{i_{e},j}\left(x\right)+\delta ,\left[p_{\min},p_{\max}\right]\right),$$
set $P_{i_{e},j}\left(1-x\right)=1-P_{i_{e},j}\left(x\right)$, and reconstruct $\left(\alpha_{i_{e},j},\beta_{i_{e},j}\right)$.  7:     AEC (non-elite): for each $i\neq i_{e}$, compute $r_{i}$ and $\Delta \theta_{i}$ according to Equations (11) and (12).  8:     For each binary gene position *j*, determine $\theta_{i,j}$ according to Equation (13), apply the norm-preserving probability-amplitude update in Equation (14), and update $P_{i,j}\left(0/1\right)$.  9:     Mutation: with mutation rate $p_{m}$, perform probability-amplitude swapping $\alpha_{i,j}↔\beta_{i,j}$, which corresponds to exchanging $P_{i,j}\left(0\right)↔P_{i,j}\left(1\right)$. 10:**end for** 11:**return **$g^{\mathrm{best}},\Theta^{\mathrm{best}}$.

## 4. Experimental Results and Analysis

This section evaluates the proposed SPA-QNAS under benchmark-consistent experimental settings. Using the same search space, circuit template, and resource budget as EQNAS, we examine whether the proposed search-dynamics enhancement improves search efficiency, update stability, and final classification performance. Experiments are conducted on the MNIST and Warship datasets. We first introduce the benchmark settings, then isolate the effects of SPE and AEC, and finally compare SPA-QNAS with the EQNAS baseline and a representative cross-paradigm QNAS method.

### 4.1. Experimental Protocol and Benchmark Settings

**Figure 5:**
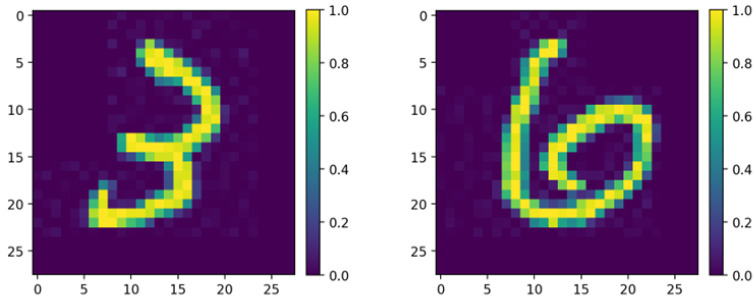
Examples of the digits 3 and 6 in the MNIST dataset.

**Figure 6:**
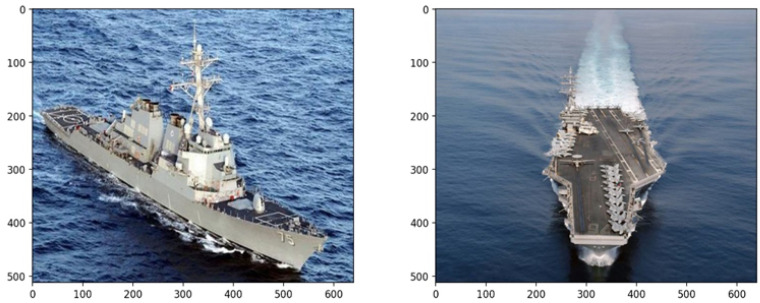
Examples of Burke and Nimitz in the Warship dataset.

Experiments are conducted on two benchmark datasets: MNIST (digits 3 vs. 6) and the Warship ship-recognition dataset (Burke vs. Nimitz). MNIST example images are shown in Figure 5. For Warship, we adopt the same benchmark setting as the original EQNAS study [25] and consider a two-class image classification task consisting of Burke and Nimitz ship images. The dataset contains 400 training images and 150 test images. Example images are shown in Figure 6. For both datasets, the preprocessing pipeline includes grayscale conversion, pixel normalization, and resizing to 8×8 to match the qubit-lattice encoding, while ensuring non-overlapping training and test splits.

**Figure 7:**
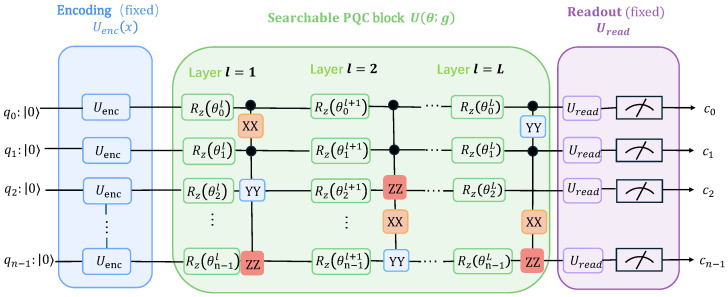
Explicit schematic of the searchable parameterized quantum circuit used in SPA-QNAS under the EQNAS benchmark setting. The circuit consists of a fixed encoding module, a searchable PQC block with candidate operations {XX,YY,ZZ,I}, and a fixed readout module.

In the implementation, quantum circuit construction and environment simulation are performed on a classical platform. Following the EQNAS benchmark setting [25], qubit-lattice encoding is used to map the preprocessed input features into quantum-state representations. Specifically, 16 data qubits are used to encode the image information, and one additional readout qubit is measured to obtain the classification output, resulting in a 17-qubit parameterized quantum circuit. The corresponding searchable PQC structure used in SPA-QNAS is illustrated in Figure 7.

The MNIST and Warship experiments are conducted on NVIDIA GeForce RTX 2080 Ti and NVIDIA GeForce RTX 3090 GPUs (NVIDIA Corporation, Santa Clara, CA, USA), respectively, following the original EQNAS benchmark setting [25]. The use of different GPU models is consistent with the original benchmark configuration and does not introduce methodological differences between SPA-QNAS and EQNAS. The parameter training uses the Adam optimizer with a learning rate of 0.001.

To ensure direct comparability with the EQNAS baseline [25], we adopt the same search budget and training configuration during the architecture-search stage. The population size is set to N=10, and the maximum number of generations is $G_{\max}=10$. The search epochs for MNIST and Warship are 3 and 10, respectively, while the final training epochs are 10 and 20, respectively. The batch sizes are 32 and 20, respectively, as listed in Table 3.

The dataset setting, circuit scale, and experimental protocol of SPA-QNAS are kept consistent with those of EQNAS, including the search budget, circuit configuration, and training settings, so that any observed performance difference can be attributed to the proposed search-dynamics enhancement rather than changes in resource scale or optimization conditions. For completeness, the hyperparameter configuration used in the evolutionary search is summarized in Table 4. All hyperparameters listed in Table 4 correspond to the actual configurations used in the reported experiments. Since each candidate gate is represented by a two-bit binary encoding, the effective genome length is 64. Unless otherwise specified, the two datasets share the same architecture-search settings.

### 4.2. Mechanism Isolation Study

To isolate the respective contributions of the two proposed mechanisms, we conduct a component-wise study based on the EQNAS benchmark. Specifically, we evaluate the baseline EQNAS, EQNAS with only the SPE strategy enabled (EQNAS + SPE), EQNAS with only the AEC mechanism enabled (EQNAS + AEC), and the complete SPA-QNAS framework. Except for the enabled mechanism, the search space, training configuration, and evaluation protocol are kept identical across all compared methods. Classification accuracy is used to measure final performance, and the reduction ratio of total runtime $R_{\mathrm{time}}$ is defined to quantify the time-efficiency improvement:

(20) $$R_{\mathrm{time}}=\frac{T_{\mathrm{base}}-T_{\mathrm{target}}}{T_{\mathrm{base}}}\times 100\%.$$

Here, $T_{\mathrm{base}}$ and $T_{\mathrm{target}}$ denote the total runtime of the baseline method and the compared method, respectively. The runtime reduction ratio is an empirical efficiency metric calculated from measured wall-clock runtimes rather than a fitted quantity; therefore, it does not involve an analytical error term.

When only SPE is introduced, the total runtime decreases substantially. On the Warship dataset, the runtime is reduced from 5:27:23 to 4:20:25, corresponding to a reduction of 20.45%. On MNIST, the runtime decreases from 27:14:32 to 7:44:23, corresponding to a reduction of 71.59%. In terms of accuracy, MNIST decreases slightly from 97.45% to 97.34%, whereas Warship increases from 82.66% to 83.33%. These results indicate that SPE, by reinforcing elite structural decisions through probability shaping, can bias the sampling distribution toward high-fitness regions and improve search efficiency. However, without adaptive alignment control, such distribution bias may introduce a certain risk of premature convergence on some tasks, which can affect the final accuracy.

When only AEC is introduced, classification performance improves steadily. Specifically, the accuracy on MNIST increases from 97.45% to 98.87%, and the accuracy on Warship increases from 82.66% to 83.33%. Regarding time efficiency, the runtime on MNIST decreases from 27:14:32 to 7:35:57, a reduction of 71.22%, while the runtime on Warship decreases from 5:27:23 to 4:40:31, a reduction of 14.31%. This behavior can be explained by the fitness-gap-based adaptive rotation mechanism in Equations (13) and (14). In particular, individuals with larger fitness gaps receive larger rotation updates, allowing low-quality structures to receive stronger alignment updates toward the elite structural pattern, while individuals closer to the elite are updated more conservatively. As a result, AEC reduces ineffective exploration in low-fitness regions and enhances population alignment toward high-fitness regions. This leads to more stable evolutionary dynamics and indirectly shortens the search process, thereby improving both final accuracy and overall search efficiency.

When both SPE and AEC are enabled, the resulting method corresponds to the complete SPA-QNAS framework. The method achieves the best overall performance in terms of both accuracy and time efficiency. On MNIST, the accuracy reaches 99.42% with a total runtime of 7:34:03, yielding a reduction of 72.22% relative to the baseline. On Warship, the accuracy increases to 85.33%, and the runtime is reduced to 3:59:33, corresponding to a reduction of 26.83%. Overall, SPE plays a major role in establishing an elite reference and enhancing probability concentration, while AEC effectively alleviates premature convergence tendencies through population alignment and disturbance suppression. Their combination balances rapid distribution concentration (SPE) and controlled population alignment (AEC), enabling the search process to achieve improved final classification performance while maintaining high efficiency. The ablation results are summarized in Table 5.

### 4.3. Comparative Analysis of Search and Classification Performance

To validate the effectiveness of the proposed SPA-QNAS method, we conduct comparative experiments on the MNIST and Warship datasets under the same experimental conditions and training settings as the EQNAS baseline. In addition, we include QuantumNAS [6] as a representative cross-paradigm QNAS reference.

#### 4.3.1. Comparison of Image Classification Performance

On MNIST, SPA-QNAS achieves a classification accuracy of 99.42%, improving upon EQNAS by 1.97 percentage points. On Warship, SPA-QNAS attains an accuracy of 85.33%, outperforming EQNAS by 2.67 percentage points. Moreover, SPA-QNAS achieves consistently higher classification accuracy than QuantumNAS on both datasets. The comparative classification results are summarized in Table 6.

**Figure 8:**
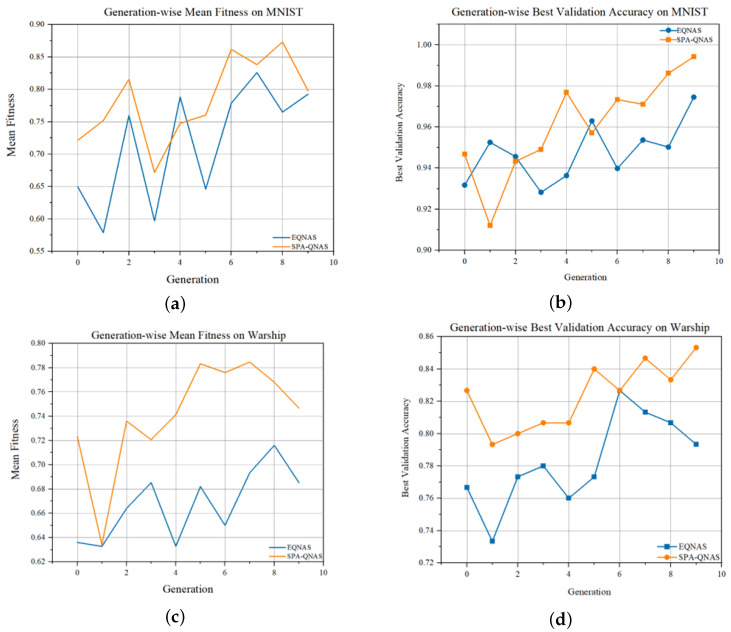
Search-phase performance comparison between EQNAS and SPA-QNAS on the MNIST and Warship datasets: (**a**) generation-wise mean fitness on MNIST; (**b**) generation-wise best validation accuracy on MNIST; (**c**) generation-wise mean fitness on Warship; (**d**) generation-wise best validation accuracy on Warship.

The search-phase performance curves are shown in Figure 8. Figure 8a and Figure 8b show the generation-wise mean fitness and best validation accuracy on MNIST, respectively, while Figure 8c and Figure 8d show the corresponding curves on Warship. Since QuantumNAS follows a supernet-based joint search paradigm and does not report generation-wise statistics in an evolutionary sense, Figure 8 only compares EQNAS and SPA-QNAS within the same evolutionary search framework.

#### 4.3.2. Convergence and Stability Analysis During the Parameter-Training Stage

**Figure 9:**
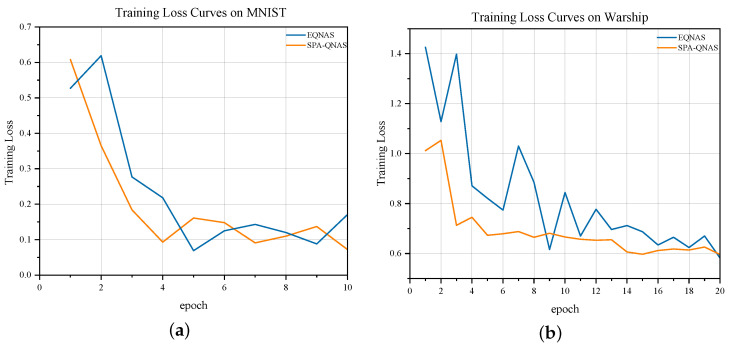
Final training loss curves of EQNAS and SPA-QNAS: (**a**) MNIST; (**b**) Warship.

Figure 9a and Figure 9b show the loss curves during the final training stage on the MNIST and Warship datasets, respectively. On MNIST, SPA-QNAS exhibits a faster loss decay in the early training stage and maintains a lower loss level in the later stage. On Warship, the loss curve of SPA-QNAS is overall smoother, with smaller oscillations during training.

This improvement in stability may be attributed to the disturbance-suppression effect of the AEC mechanism. For low-performing individuals that are far from the elite structure, AEC adaptively adjusts the rotation step size of probability-amplitude updates, thereby reducing excessive perturbations from low-quality structures to the population-level QPV distribution. As a result, the selected architectures exhibit smoother training behavior and improved robustness during the final parameter-training stage.

#### 4.3.3. Model Complexity Analysis

To ensure a fair evaluation under a fixed quantum resource budget, SPA-QNAS and the EQNAS baseline are constrained to use the same circuit scale. As shown in Table 7, both methods use the same number of qubits, quantum gates, and trainable parameters on the MNIST and Warship datasets. Therefore, the performance improvement of SPA-QNAS is not achieved by increasing the number of qubits, quantum gates, or trainable parameters, but instead results from the proposed enhancements to the search dynamics, including SPE-based structural probability enhancement and AEC-based adaptive QPV updates.

The purpose of this analysis is not to modify the final circuit complexity, but to verify whether improving evolutionary search dynamics can enhance search efficiency and final classification performance under the same quantum resource budget as EQNAS. As shown in Table 7, SPA-QNAS achieves improved performance without introducing additional quantum resources or increasing the complexity of the final selected architecture.

#### 4.3.4. Brief Comparison with Representative Quantum Neural Architecture Search Methods

To clarify the methodological positioning of SPA-QNAS in existing studies on quantum neural architecture search, we select representative methods from different paradigms, including EQNAS [25], QuantumNAS [6], QWAS [20], and oDARTS [22], and conduct a mechanism-level comparison, as summarized in Table 8. Since these methods differ substantially in search space definitions, encoding schemes, training budgets, and evaluation protocols, cross-paper numerical comparisons are not strictly fair. Therefore, this section focuses on a qualitative comparison along three dimensions: search paradigm, guidance mechanism, and control strategy. Quantitative performance comparisons between SPA-QNAS and EQNAS under identical experimental settings are provided in the experimental evaluation section.

As shown in Table 8, SPA-QNAS builds upon the evolutionary search framework of EQNAS and further introduces explicit elite-guided probability reinforcement through SPE and adaptive population-level alignment through AEC. Unlike QuantumNAS, QWAS, and oDARTS, which employ fundamentally different optimization paradigms, SPA-QNAS aims to improve the search dynamics of the original QEA-based framework while preserving the same search space, circuit template, training configuration, and quantum resource budget as EQNAS. Therefore, the comparison in Table 8 is intended to highlight the methodological differences among representative QNAS approaches rather than provide strictly quantitative comparisons across heterogeneous search paradigms.

#### 4.3.5. Statistical Analysis of Multiple Runs

To evaluate robustness, we conduct five independent runs using different random seeds. Table 9 reports the statistical results over five independent runs, including best accuracy, mean accuracy, standard deviation, and 95% confidence intervals.

The results show that SPA-QNAS achieves higher mean accuracy and lower variance compared with EQNAS, indicating improved stability across independent runs. In particular, the reduced standard deviation demonstrates that SPA-QNAS is less sensitive to random initialization and stochastic sampling during the evolutionary search process.

We further perform Welch’s *t*-test based on the results of five independent runs, obtaining a *p*-value of 0.198 (>0.05). This indicates that the observed improvement is not statistically significant at the 95% confidence level under the current experimental setting. Nevertheless, SPA-QNAS consistently shows a higher mean performance and reduced variance, indicating a stable improvement trend across different random seeds.

We compute the 95% confidence intervals using the Student’s *t*-distribution over five independent runs (n = 5).

### 4.4. Population Diversity Analysis

To evaluate whether the proposed SPE mechanism induces premature convergence, we analyze the population diversity throughout the evolutionary process on the Warship dataset. Population diversity is quantified by the average pairwise Hamming distance among chromosomes.

Let $P^{\left(t\right)}=\left\{g_{1}^{\left(t\right)},g_{2}^{\left(t\right)},\ldots ,g_{N}^{\left(t\right)}\right\}$ denote the population at generation *t*. The diversity is defined as:

(21) $$D^{\left(t\right)}=\frac{2}{N(N-1)}\sum_{i<j}H\left(g_{i}^{\left(t\right)},g_{j}^{\left(t\right)}\right)$$

**Figure 10:**
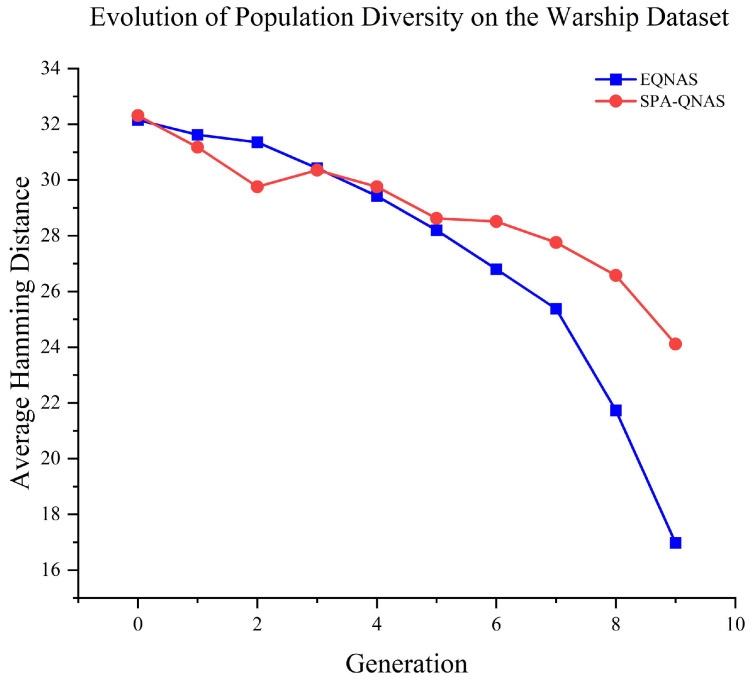
Evolution of population diversity during the evolutionary search on the Warship dataset.

As shown in Figure 10, EQNAS exhibits a faster decline in diversity, indicating premature convergence. In contrast, SPA-QNAS maintains higher diversity throughout the evolutionary process, demonstrating that SPE and AEC effectively preserve exploration capability while improving convergence efficiency.

## 5. Conclusions

This paper proposed SPA-QNAS, a search-dynamics-enhanced evolutionary QNAS framework built upon the EQNAS benchmark, to address slow distribution concentration and insufficient update adaptivity in image classification tasks. Unlike methods that improve performance by increasing circuit scale or quantum resource consumption, SPA-QNAS focuses on enhancing the evolutionary search dynamics under a fixed quantum resource budget. The proposed method integrates SPE and AEC into a QPV-driven evolutionary search loop. SPE reinforces the contemporary elite structure through bounded probability shaping, thereby promoting the concentration of the structural sampling distribution toward high-fitness regions. AEC uses the elite structure as a reference and adaptively adjusts the rotation update strength of non-elite individuals according to fitness feedback, which reduces ineffective disturbances and improves update stability. Together, SPE and AEC form a closed-loop search enhancement mechanism that connects structural sampling, fitness evaluation, and QPV evolution. Experimental results on the MNIST and Warship datasets demonstrate the effectiveness of SPA-QNAS. Under the same 17-qubit circuit scale and identical training configuration as EQNAS, SPA-QNAS achieves classification accuracies of 99.42% and 85.33% on the MNIST and Warship datasets, respectively, without increasing the number of qubits, quantum gates, or trainable parameters. In addition, statistical analysis over five independent runs demonstrates that SPA-QNAS achieves higher mean accuracy and substantially lower variance than EQNAS, indicating improved robustness across different random seeds. Population diversity analysis further shows that the proposed SPE and AEC mechanisms effectively preserve exploration capability while alleviating premature convergence during the evolutionary process.These results indicate that improving QPV-based search dynamics is an effective approach for enhancing the stability, robustness, and search capability of evolutionary QNAS under fixed quantum resource constraints.

## Figures and Tables

**Table 1:** List of abbreviations and acronyms used in this paper.

Acronym	Full Name
CNAS	Classical Neural Architecture Search
QNAS	Quantum Neural Architecture Search
QNN	Quantum Neural Network
PQC	Parameterized Quantum Circuit
QEA	Quantum Evolutionary Algorithm
QPV	Quantum-Inspired Probability-Amplitude Vector
EQNAS	Evolutionary Quantum Neural Architecture Search
SPA-QNAS	Structural Probability Adaptive Quantum Neural Architecture Search
SPE	Structural Probability Enhancement
AEC	Adaptive Evolutionary Control
NISQ	Noisy Intermediate-Scale Quantum

**Table 2:** Adaptive evolutionary control rotation direction rules (based on the elite bit and current bit).

Elite Bit $(g_{i_{e}}{)}_{j}$	Current Bit $(g_{i}{)}_{j}$	$\theta_{i,j}$
0	0	0
1	1	0
0	1	$-\Delta \theta_{i}$
1	0	$+\Delta \theta_{i}$

**Table 3:** Experimental environment and training configuration.

Item	MNIST	Warship
GPU	NVIDIA GeForce RTX 2080 Ti	NVIDIA GeForce RTX 3090
Optimizer	Adam	Adam
Learning rate (Adam)	0.001	0.001
Framework	MindSpore 1.8.1/MindQuantum 0.7.0	MindSpore 1.8.1/MindQuantum 0.7.0
Qubits	17 (16 Data + 1 Readout)	17 (16 Data + 1 Readout)
Epochs (search)	3	10
Batch size	32	20
Epochs (final)	10	20
Encoding	Qubit-lattice	Qubit-lattice
Loss	Cross-Entropy	Cross-Entropy

**Table 4:** Hyperparameter configuration for evolutionary search in SPA-QNAS.

Parameter	Symbol	Value
Population size	*N*	10
Max generations	$G_{\max}$	10
Genome length	*L*	64
Rotation angle	$\left(\theta_{\min},\theta_{\max}\right)$	(0.005π,0.05π)
SPE step	δ	0.1
Probability bounds	$\left[p_{\min},p_{\max}\right]$	[0.02,0.98]
Mutation rate (population/gene)	–	0.01/0.002
Candidate gate set	–	{XX,YY,ZZ,I}

**Table 5:** Ablation experiment results.

Methods	MNIST Acc	MNIST Time (h:m:s)	Warship Acc	Warship Time (h:m:s)
EQNAS	97.45%	27:14:32	82.66%	5:27:23
EQNAS + SPE	97.34%	7:44:23	83.33%	4:20:25
EQNAS + AEC	98.87%	7:35:57	83.33%	4:40:31
SPA-QNAS	99.42%	7:34:03	85.33%	3:59:33

**Table 6:** Comparative classification performance on MNIST and Warship.

Model	MNIST Acc	Warship Acc
EQNAS	97.45%	82.66%
SPA-QNAS	99.42%	85.33%
QuantumNAS	86.67%	66.67%

**Table 7:** Model complexity comparison between EQNAS and SPA-QNAS under the same quantum resource budget.

Dataset	Method	Qubits	Gates	Parameters
MNIST	EQNAS	17	30	30
MNIST	SPA-QNAS	17	30	30
Warship	EQNAS	17	30	30
Warship	SPA-QNAS	17	30	30

**Table 8:** Mechanism-level comparison of representative quantum neural architecture search methods.

Method	Search Paradigm	Guidance Mechanism	Control Strategy
EQNAS [25]	Evolutionary (QEA)	No explicit probability enhancement	Fixed-step rotation (QPV)
QuantumNAS [6]	Supernet + joint search	Noise-aware	Parameter-inheritance-based evaluation + evolutionary search
QWAS [20]	Qubit-wise staged search	MCTS	Stage-wise structural updates
oDARTS [22]	Differentiable search	Continuous relaxation	Density-matrix gradient optimization
SPA-QNAS	Evolutionary (QEA)	Elite structural guidance via SPE	AEC-based adaptive non-elite QPV alignment

**Table 9:** Comparison of EQNAS and SPA-QNAS on the Warship dataset over five independent runs (Best, Mean, Std, and 95% CI).

Method	Best	Mean	Std	95% CI
EQNAS	0.8266	0.8187	0.0191	[0.795, 0.842]
SPA-QNAS	0.8533	0.8320	0.0056	[0.825, 0.839]

## Data Availability

The MNIST dataset used in this study is publicly available. The Warship dataset is adopted from the benchmark setting of the EQNAS study. The experimental logs and detailed implementation settings are available from the corresponding author upon reasonable request. Due to an approved patent protecting the proposed SPA-QNAS framework, the source code is not publicly released at this stage. However, sufficient algorithmic details and pseudo-code are provided in the manuscript to ensure reproducibility of the proposed method.
